# Plasmonic Catalysis for Controlling Selectivity in the Hydrogenation of Cinnamaldehyde to Propylbenzene Under Visible‐Light Irradiation

**DOI:** 10.1002/cssc.202501054

**Published:** 2025-08-12

**Authors:** Sana Frindy, Shiqi Wang, Sam Sullivan–Allsop, Rongsheng Cai, Thomas J. A. Slater, Sarah J. Haigh, Pedro H. C. Camargo

**Affiliations:** ^1^ Department of Chemistry University of Helsinki A.I. Virtasen aukio 1, PO Box 55 FIN‐0014 Helsinki Finland; ^2^ Department of Materials University of Manchester Manchester M13 9PL UK; ^3^ Cardiff Catalysis Institute School of Chemistry Cardiff University Cardiff CF10 3AT UK

**Keywords:** cinnamaldehyde hydrogenation, density functional theories, plasmonic catalysis, propylbenzene, selectivity control, visible‐light irradiations

## Abstract

The selective hydrogenation of α,β‐unsaturated aldehydes, such as cinnamaldehyde (CAL), into value‐added aromatic hydrocarbons like propylbenzene (PPR) remains a formidable challenge due to competing C=C and C=O hydrogenation pathways. Here, a plasmon‐enhanced catalytic strategy employing Au@Au_3_Pd core–shell nanoparticles supported on silica is reported. The catalyst features a plasmonic Au core and a 1 nm Au_3_Pd alloyed shell (25 at% Pd), enabling light‐driven modulation of reaction selectivity. Under visible‐light irradiation, the catalyst achieves complete CAL conversion with a ≈34% yield of PPR, corresponding to a 7.7‐fold enhancement in turnover frequency relative to dark conditions. Density functional theory calculations reveal that interfacial electronic coupling between the Au core and Pd‐rich shell upshifts the Pd *d*‐band center and enhances charge transfer, promoting both C=C and C=O hydrogenation steps followed by hydrogenolysis to PPR. This study demonstrates a robust approach to overcome selectivity limitations in multifunctional molecule hydrogenation by harnessing localized surface plasmon resonance effects. The insights gained offer a foundation for the rational design of light‐responsive bimetallic catalysts for selective and sustainable transformations.

## Graphical abstract

A plasmonic Au@Au_3_Pd catalyst that integrates visible‐light‐driven hot electron generation with bimetallic electronic tuning to steer the selective hydrogenation of cinnamaldehyde toward propylbenzene is reported. Localized surface plasmon resonance excitation modulates reaction pathways on tailored Au–Pd interfaces, enabling product selectivity under mild conditions.

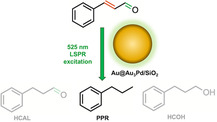

## 1 Introduction

**Scheme 1:**
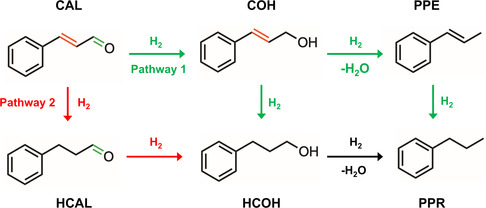
Hydrogenation routes for cinnamaldehyde (CAL) to propylbenzene (PPR). The scheme illustrates two distinct pathways dictated by initial hydrogenation selectivity. The upper pathway, initiated by C=O hydrogenation, proceeds through cinnamyl alcohol (COH) and either hydrogenolysis to 1‐phenylpropene (PPE) followed by C=C hydrogenation or direct conversion to hydrocinnamyl alcohol (HCOH), which subsequently undergoes hydrogenolysis to PPR. The lower pathway, initiated by C=C hydrogenation, proceeds through hydrocinnamaldehyde (HCAL) and hydrocinnamyl alcohol (HCOH), culminating in hydrogenolysis to PPR.

Propylbenzene (PPR), a valuable building block in the synthesis of pharmaceuticals, agrochemicals, and specialty materials, can be accessed through the catalytic hydrogenation of cinnamaldehyde (CAL).^[^ ^1^ ^]^ This reaction exemplifies the broader field of α,β‐unsaturated aldehyde hydrogenation, a cornerstone of modern synthesis underpinning the production of fine chemicals, pharmaceuticals, and commodity additives.^[^ ^1^, ^2^ ^]^ Despite advancements in both homogeneous and heterogeneous catalytic methods, achieving high selectivity for fully hydrogenated products under mild conditions remains elusive.^[^ ^3^, ^4^, ^5^ ^]^ Conventional heterogeneous catalysts based on transition metals often fail to deliver the requisite selectivity for PPR.^[^ ^1^, ^4^, ^6^, ^7^, ^8^ ^]^ This limitation arises from the structural complexity of CAL, which contains two readily reducible bonds (C=C and C=O). These bonds frequently generate partially hydrogenated products, such as hydrocinnamaldehyde (HCAL), cinnamyl alcohol (COH), and hydrocinnamyl alcohol (HCOH) (**Scheme** **1**).^[^ ^7^, ^9^ ^]^ While efforts have targeted better control over these intermediates,^[^ ^1^, ^3^, ^8^, ^10^, ^11^, ^12^ ^]^ a key challenge persists: designing catalysts that not only activate both C=C and C=O groups efficiently but also direct the overall transformation toward propylbenzene.

Several strategies have been proposed to produce catalysts that can overcome these selectivity hurdles, including alloying, nanostructuring, single‐atom materials, and plasmonic enhancement.^[^ ^1^, ^3^, ^11^, ^12^, ^13^, ^14^, ^15^, ^16^, ^17^ ^]^ In particular, plasmonic‐based catalysts have garnered attention for utilizing localized surface plasmon resonance (LSPR) under visible‐light irradiation, thus enabling milder process conditions and new routes to control reaction selectivity compared to purely thermal methods.^[^ ^18^, ^19^, ^20^, ^21^, ^22^, ^23^, ^24^, ^25^, ^26^, ^27^ ^]^ Various plasmonic‐catalytic nanoparticles (NPs), including core–shell, core–satellite, and alloy morphologies, exploit LSPR‐generated hot carriers (and localized heating) to drive specific reactions at adjacent catalytic sites.^[^ ^21^, ^22^, ^26^, ^28^, ^29^, ^30^, ^31^ ^]^ Nevertheless, precise modulation of product selectivity in such hybrid plasmonic architectures remains challenging.^[^ ^25^, ^32^ ^]^

Here, we employ core‐alloyed shell Au@Au_3_Pd NPs with an optimized overall 3 at% Pd loading^[^ ^33^ ^]^ (25 at% of Pd on a 1 nm shell) to demonstrate that plasmonic catalysis can simultaneously achieve high CAL conversion and promote reaction selectivity toward PPR under mild, visible‐light‐driven conditions. Our approach leverages the strong LSPR of Au to generate hot electrons under visible‐light irradiation, which are then efficiently transferred to Pd‐based active sites to increase selectivity to PPR via CAL hydrogenation. This enables the combination of strong visible‐light absorption and the electronic synergy arising from the Au–Pd interface. Through experimental studies and density functional theory (DFT) calculations, we unravel how plasmon‐assisted charge transfer steers the reaction toward complete hydrogenation, overcoming the intrinsic selectivity limitations commonly encountered in CAL hydrogenation. Our findings shed light on a promising avenue for integrating plasmonic enhancement into heterogeneous catalysis and provide a promising strategy for designing plasmonic catalysts for sustainable chemical production.

## 2 Results and Discussion

**Figure 1:**
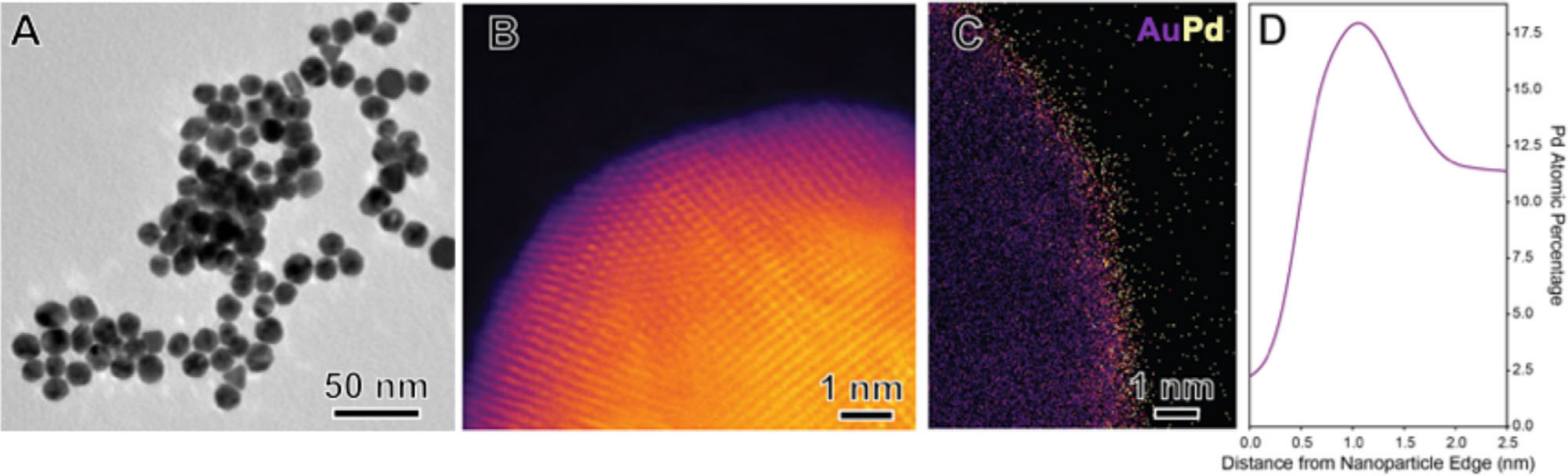
Structural and local elemental characterization of Au@Au_**3**_Pd NPs. A) TEM image. B) STEM‐HAADF image of the surface of a single NP. C) STEM‐EDX elemental mapping showing the Pd distribution. D) Radially averaged EDX line profiles from NPs in (C), demonstrating Pd enrichment at the NP surface; the full width at half maximum of the Pd surface peak is 0.9 nm. The Au@Au_3_Pd NPs are 16 ± 3 nm in diameter.

The key to achieving high PPR selectivity from CAL hydrogenation (Scheme 1) lies in steering the reaction along initial hydrogenation pathways while favoring subsequent hydrogenation and hydrogenolysis steps. To this end, we hypothesize that visible‐light‐induced LSPR can synergistically boost catalytic activity and, crucially, direct product selectivity, thus enabling both C=C and C=O hydrogenation as well as C—OH hydrogenolysis leading to PPR. Our approach centers on the recent development of Au@Au_3_Pd (97 at% Au and 3 at% Pd) NPs, composed of an Au core enveloped by a dilute Au_3_Pd alloy shell ≈1.0 nm thick and containing 25 at% Pd at the shell (Au@Au_3_Pd, **Figure** **1**).^[^ ^33^ ^]^ These NPs offer i) efficient plasmonic response under visible light, attributed to low Pd content and the bimetallic alloy surface;^[^ ^34^ ^]^ ii) synergistic catalytic activity imparted by the bimetallic Au_3_Pd alloy compared to Pd metal, known to promote beneficial electronic effects; and iii) efficient active metal utilization enabled by the low Pd loading.

**Figure 2:**
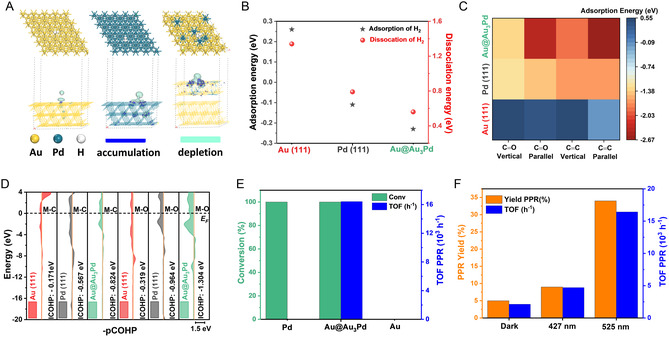
Computational and experimental evidence for plasmon‐enhanced cinnamaldehyde hydrogenation. A) CDD analysis of H_2_ adsorption on Au, Pd, and Au@Au_3_Pd (111) surfaces, visualizing electronic perturbation. B) Energetics of H_2_ adsorption and dissociation on various sites, revealing enhanced H_2_ activation on Au@Au_3_Pd. C) CAL adsorption energies on Au, Pd, and Au@Au_3_Pd, indicating preferred binding sites. D) COHP analysis of metal–cinnamaldehyde interactions (M—C and M—O bonds), elucidating bonding nature in different configurations. E) CAL conversion and TOF values calculated for PPR formation over Au/SiO_2_, Pd/SiO_2_, and Au@Au_3_Pd/SiO_2_ under 525 nm irradiation conditions. F) CAL conversion under dark conditions and when irradiated with 427 nm and 525 nm light. Reaction time corresponded to 24 h. The irradiance of the 427 nm LED was 126.1 mW cm^−2^, and the irradiance of the 525 nm LED was 59.5 mW cm^−^^2^. Despite the higher intensity of the 427 nm light, the enhancement under 525 nm excitation was significantly greater, indicating a dominant role of resonant plasmonic excitation.

DFT calculations provide insight into the CAL hydrogenation behavior and reaction pathways on Au, Pd, and bimetallic Au@Au_3_Pd catalytic surfaces. As the (111) plane is typically the most stable and catalytically relevant facet, Au(111), Pd(111), and Au@Au_3_Pd(111) models were constructed (Figure S1, Supporting Information), with the alloy having a Pd composition at the surface of 25 at% consistent with scanning transmission electron microscope‐energy‐dispersive X‐ray (STEM‐EDX) measurements (Pd randomly distributed into the Au layer). The stable adsorption and spontaneous dissociation of H_2_ are essential prerequisites for effective hydrogenation; therefore, the energetics of H_2_ adsorption were examined on the constructed surface models. **Figure** **2**A shows the optimized adsorption configurations and charge density difference (CDD) plots, which reveal that H_2_ induces a more pronounced charge redistribution on Au_3_Pd than on the monometallic Au or Pd surfaces.^[^ ^35^ ^]^ Mulliken charge analysis (Figure S2, Supporting Information) confirms that both Au@Au_3_Pd and Pd display stronger charge‐transfer characteristics to adsorbed H_2_ as compared to Au. As illustrated in Figure 2B, the adsorption energy of H_2_ on Au@Au_3_Pd is notably more negative than that on Au or Pd, indicating a more stable adsorbate–surface interaction.^[^ ^36^ ^]^ Furthermore, the Au@Au_3_Pd model exhibits the lowest energy barrier for H_2_ dissociation, indicating that H_2_ cleavage is favored at the bimetallic interface.^[^ ^1^ ^]^ Pd(111) can also adsorb and dissociate H_2_ while Au(111) shows only limited activity for this process. These results suggest that a pure Au surface will not enable H_2_ activation, while Au@Au_3_Pd and Pd surfaces do, thus enabling subsequent hydrogenation reactions.

The subsequent hydrogenation selectivity step depends on the adsorption configuration of CAL on the catalyst surface. Thus, CAL adsorption energies were calculated on the Au(111), Pd(111), and Au@Au_3_Pd(111) models for both C=O‐ and C=C‐bound configurations (Figure S3 and S4, Supporting Information). As summarized in Figure 2C, parallel adsorption modes generally possess more negative adsorption energies than their vertical counterparts. Notably, Au@Au_3_Pd displays a significantly stronger affinity for CAL than either Au or Pd, with comparable energies for adsorption through C=O and C=C bonds (−2.42 eV and −2.66 eV, respectively). This finding indicates that Au_3_Pd can potentially enable CAL hydrogenation through both the carbonyl and the α,β‐unsaturated bond (favoring both pathways 1 and 2 from Scheme 1).^[^ ^37^ ^]^ In contrast, on Pd(111), adsorption through the C=C group is more favorable relative to the C=O group, suggesting a higher propensity for hydrogenation at the C=C bond (pathway 2 in Scheme 1). Meanwhile, Au(111) exhibits much weaker interaction energies across all configurations, underscoring its comparatively limited catalytic activity.

To further quantify the strength of CAL–surface interactions, the crystal orbital Hamilton population (COHP) and the corresponding integral COHP values for the M—C and M—O bonds (where M is the surface metal site) were calculated, as shown in Figure 2D. These metrics confirm that Au@Au_3_Pd establishes the strongest bonds with both the carbon and oxygen sites of CAL,^[^ ^38^ ^]^ surpassing Au and Pd. These DFT results highlight that a Au@Au_3_Pd catalytic surface not only promotes H_2_ dissociation but also favorably adsorbs CAL via both the C=O and C=C groups. Adsorption via both double bonds facilitates and enables multiple hydrogenation pathways, rendering Au@Au_3_Pd an attractive candidate for the full CAL hydrogenation with the potential for enhanced selectivity to PPR.^[^ ^18^, ^39^ ^]^

Guided by these results, Au@Au_3_Pd NPs were supported on silica (Au@Au_3_Pd /SiO_2_) and employed as catalysts for CAL hydrogenation. SiO_2_ was selected as an inert support to minimize or eliminate any potential contributions from the support itself, thereby enabling a more accurate assessment of the intrinsic catalytic properties of the core–shell NPs. Monometallic Au/SiO_2_ and Pd/SiO_2_ samples were also prepared as references. Figure 2E and Table S1, Supporting Information, summarize the CAL conversion and turnover frequency (TOF) values for propylbenzene (PPR) formation under 525 nm light irradiation (the extinction spectra of Au@Au_3_Pd NPs are shown in Figure S5, Supporting Information). Neither Au/SiO_2_ nor Pd/SiO_2_ produced detectable amounts of PPR. Pd/SiO_2_ fully converted CAL, and the products corresponded to partially hydrogenated HCAL (81%) and HCOH (19%). Thus, both monometallic Au and Pd catalysts lacked the necessary combination of activity and selectivity for complete CAL hydrogenation to PPR under our employed conditions, consistent with our DFT predictions of surface adsorption. By contrast, the Au@Au_3_Pd/SiO_2_ sample achieved 100% CAL conversion, producing a PPR yield of 34% with a TOF of 1.64 × 10^4^ h^−1^.

To further validate the role of plasmon excitation in the hydrogenation activity of Au@Au_3_Pd/SiO_2_, we conducted control experiments both in the dark and under varying irradiation wavelengths. Under dark conditions, although 100% of the CAL was converted, the PPR yield was only 5% (PPR TOF of 2.13 × 10^3^ h^−1^, Figure 2F). This data indicate that plasmonic excitation under visible‐light irradiation enables control over the reaction selectivity, increasing the formation of PPR. Our data indicate that LSPR excitation leads to 6.8‐ and 7.7‐fold increase in PPR yield and TOF, respectively. Changing the light irradiation wavelength to 427 nm, which is outside of the maximum of the LSPR band (Figure S5, Supporting Information), resulted in lower enhancement as compared to 525 nm excitation despite its higher light intensity. These findings confirm the wavelength‐dependent plasmonic effect and highlight the role of LSPR rather than light intensity alone as the primary factor driving the observed selectivity.^[^ ^18^, ^21^, ^31^, ^39^ ^]^ This wavelength‐dependent enhancement is a well‐established signature of plasmonic catalysis driven by hot charge carriers. The fact that 525 nm excitation (resonant with the Au@Au_3_Pd LSPR band) leads to a 7.7‐fold increase in TOF compared to dark conditions, while 427 nm off‐resonance excitation results in significantly lower enhancement, supports the conclusion that the observed selectivity shift arises predominantly from LSPR‐generated hot electrons. A purely photothermal mechanism would be expected to correlate with overall light intensity, yet in our case, the higher intensity 427 nm irradiation yields lower catalytic enhancement, further ruling out heating as the dominant factor. Thus, our data strongly support a plasmonic mechanism whereby hot carriers generated at the Au core transfer to the catalytically active Pd shell, selectively modulating reaction pathways, although localized heating can also contribute to increased reaction rates under plasmonic excitation.^[^ ^18^, ^21^, ^31^, ^39^ ^]^

Lowering the Pd content from 3 at% in the Au@Au_3_Pd/SiO_2_ NPs to 1.5 and 0.3 reduced the corresponding PPR TOFs to 3.6 × 10^3^ h^−1^ (9% yield) and 1.2 × 10^3^ h^−1^ (2% yield), respectively (Table S1, Supporting Information). This reduced PPR selectivity at lower Pd content likely stems from a reduced number of accessible neighboring Pd sites on the NP surface, hindering the parallel adsorption of CAL (via both C=O and C=C bonds, Figure S3, Supporting Information) and thereby reducing the likelihood of hydrogenation steps via both C=O and C=C groups that can favor PPR formation. These results underscore that precisely tuning the Pd composition is crucial for enhancing hydrogenation activity and CAL adsorption to improve PPR selectivity. While we did not explore higher Pd loadings, we anticipate, based on plasmonic design principles and our mechanistic insights, that further increasing Pd content would lead to LSPR damping and a shift in catalytic behavior toward that of bulk Pd, which we have shown lacks selectivity for PPR. This suggests that the Au@Au_3_Pd composition represents a finely tuned balance between maximizing plasmonic enhancement and maintaining the optimal density of active bimetallic sites for selective catalysis.

Au/SiO_2_ and Pd/SiO_2_ showed no selectivity toward PPR under light and are not expected to perform differently under dark conditions. Similarly, the bimetallic Au@Au_7_Pd and Au@Au_39_Pd systems yielded only 9% and 2% PPR under optimal illumination (Table S1, Supporting Information), indicating that their performance would further decline in the dark due to insufficient active site density and lack of plasmonic excitation. Thus, Figure 2F, which compares the optimal Au@Au_3_Pd catalyst under light versus dark conditions, remains the most direct and informative demonstration of plasmonic enhancement. Collectively, these comparisons allow us to isolate the LSPR effect without requiring further dark‐condition experiments.

**Figure 3:**
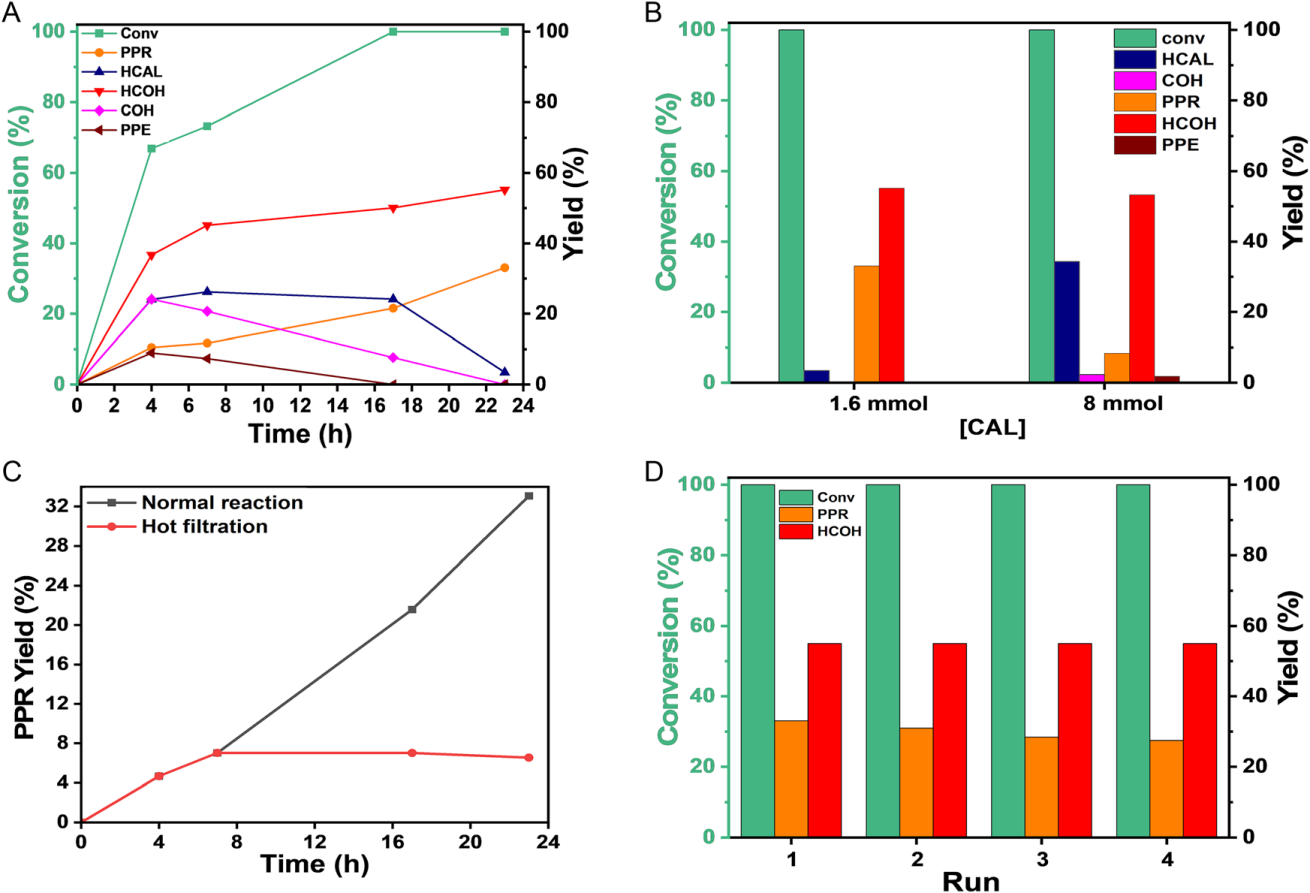
High efficiency and stability of Au@Au_**3**_Pd/SiO_2_ for plasmonic‐catalytic cinnamaldehyde hydrogenation. A) CAL conversion and product selectivity over reaction time. B) Effect of CAL/Pd ratio on reaction outcome, revealing optimal catalyst utilization. C) Hot filtration experiment: Temporal PPR yield for the reaction in the presence of the catalyst (black trace) and for the reaction in which the catalyst is removed at 7 h (red trace), confirming the heterogeneous catalysis process. D) Reusability of Au@Au_3_Pd/SiO_2_ catalyst, depicting conversion and product selectivity after consecutive reaction cycles. All reactions were performed under 525 nm irradiation. Reaction time corresponded to 24 h.

To gain further insights into how LSPR excitation influences reaction selectivity, CAL conversion and product distributions were monitored over time using Au@Au_3_Pd/SiO_2_ under visible‐light irradiation (Figure 3A). Full CAL conversion was achieved by 17 h, with HCOH and PPR emerging as the major products at 23 h. The PPR formation steadily increased throughout the reaction, ultimately reaching ≈34% at 23 h. Among the products, HCOH has the highest yield, rising sharply until 8 h and continuing to increase more gradually thereafter, reflecting its formation via hydrogenation of HCAL and COH. Conversely, COH and 1‐phenylpropene (PPE) peaked at 4 h, while HCAL (blue line) showed only modest changes from 4 to 17 h, indicating a slower rate of further hydrogenation compared to COH. Altogether, these time‐resolved data confirm that Au@Au_97_Pd_3_/SiO_2_ effectively mediates both C=C and C=O hydrogenation steps essential to PPR formation, resulting in multiple parallel pathways. Balancing these reaction routes and harnessing plasmonic enhancements are thus key to maximizing PPR selectivity. The strong performance of Au@Au_3_Pd/SiO_2_ underscores how carefully tuning bimetallic composition and plasmon resonance can synergistically deliver high conversion and increase selectivity for the hydrogenation of CAL to PPR. While a time‐resolved dark‐condition profile was not included here to preserve visual clarity, the dramatic enhancement in both PPR yield and TOF under 525 nm illumination relative to dark conditions is shown in Figure 2F. This light‐versus‐dark comparison directly confirms the role of plasmonic excitation in modulating product selectivity, complementing the kinetic insights provided by **Figure** **3**A. Given that the Au@Au_7_Pd and Au@Au_39_Pd catalysts displayed very low PPR yields and TOFs even under optimal illumination, their dark‐condition performance would necessarily be negligible and scientifically uninformative. We therefore focused the light–dark mechanistic comparison on the catalytically relevant Au@Au_3_Pd system, which provides the most robust evidence for plasmon‐driven selectivity.

In addition, the effect of the Pd/CAL molar ratio was examined by varying the initial CAL concentration from 1.6 mmol to 8 mmol, effectively decreasing the relative amount of Pd per substrate molecule, as shown in Figure 3B. Under these conditions, the yield of PPR dropped from 33% to 9% (Figure 3B), whereas HCOH remained at ≈55% and HCAL increased substantially from 3% to 35%. This shift in selectivity can be traced to a reduction in the hydrogen donor capacity, which describes how effectively hydrogen is supplied and transferred to the deeper hydrogenation steps required to form PPR.^[^ ^12^ ^]^ Specifically, when fewer Pd sites are available per CAL molecule, there is insufficient H_2_ dissociation and hydrogen transfer to drive the complete reaction. As a result, partially hydrogenated species such as HCAL and HCOH accumulate, causing the pathway to stall before full hydrogenolysis can occur.^[^ ^12^ ^]^ Despite the lower PPR yield at the higher CAL loading, the Au@Au_3_Pd/SiO_2_ catalyst still achieved 100% CAL conversion, demonstrating its robust catalytic activity. These findings underscore the need to carefully balance active‐site density, hydrogen donor capacity, and reaction conditions to direct selectivity toward full hydrogenation.

To confirm that the reaction proceeds via a heterogeneous pathway, hot filtration experiments were performed at 70 °C using Au@Au_3_Pd/SiO_2_ under optimal reaction conditions (Figure 3C). Once the catalyst was filtered off at ≈70% CAL conversion, no further increase in PPR yield was observed, remaining unchanged over the subsequent reaction period. This finding indicates that no significant catalytically active species leached into the liquid phase; hence, the hydrogenation of CAL proceeds via a heterogeneous mechanism. To evaluate the reusability of Au@Au_3_Pd/SiO_2_ under reaction conditions, a series of consecutive reactions was performed using the same recovered catalyst sample. After each run, the solid catalyst was recovered by centrifugation, washed with fresh isopropanol, and reused under identical conditions. As shown in Figure 3D, the catalytic activity remained stable through four consecutive cycles in terms of CAL conversion. A slight drop in the PPR yield was detected after five runs from 34% (to 27.5%). Scanning electron microscopy (SEM) images (Figure S6, Supporting Information confirmed that the morphology of Au@Au_3_Pd/SiO_2_ was largely preserved after the stability tests. The average NP diameter shifted only modestly, from 16 ± 3 nm (fresh) to 20 ± 5 nm after five catalytic cycles, with the two distributions largely overlapping within experimental error. This minor broadening confirms that the Au@Au_3_Pd/SiO_2_ catalyst retains its structural integrity and catalytic performance under the employed conditions.

**Figure 4:**
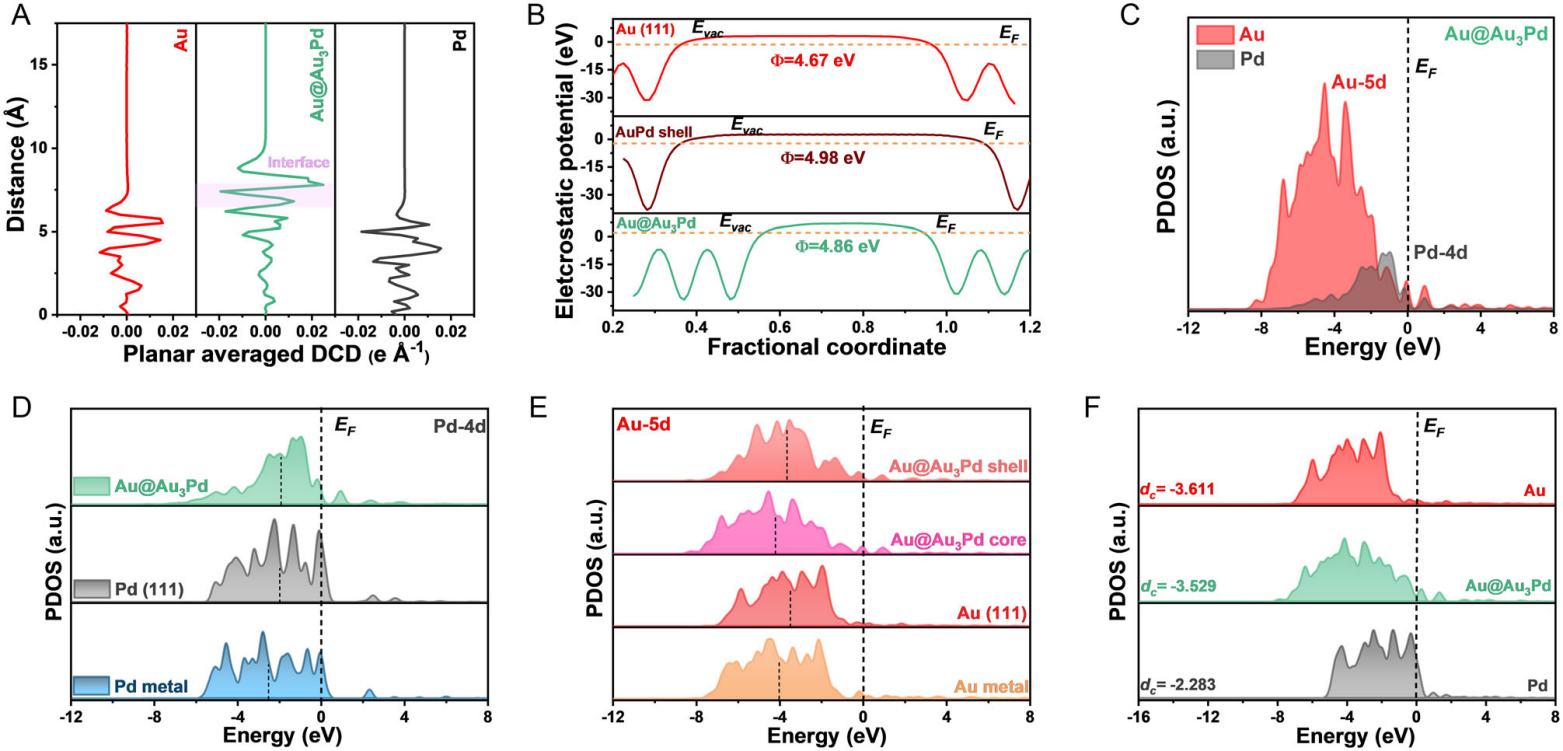
Electronic structure modulation in Au@Au_**3**_Pd NPs revealed by DFT. A) Plane‐averaged DCD profiles, demonstrating interfacial charge accumulation along the z‐axis in the Au@Au_3_Pd model compared to Au and Pd surfaces. B) Electrostatic potential for Au bulk models, Au_3_Pd alloy shell, and Au@Au_3_Pd models, revealing surface potential modifications. C) PDOS for Au@Au_3_Pd, showing the overall electronic band structure. (D and E) Site‐projected *d*‐band density of states (d‐PDOS) of D) Pd 4*d* and E) Au 5*d* orbitals within different coordination environments in Au@Au_3_Pd. F) Comparison of d‐PDOS for Au, Pd, and Au@Au_3_Pd, highlighting the electronic structure modifications induced by the core‐alloyed shell structure.

DFT calculations were subsequently employed to elucidate the electronic origins of the enhanced plasmon‐driven catalysis observed with Au@Au_3_Pd NPs. The analysis focused on how the core–shell architecture mediates electron density transfer and modifies electronic states to facilitate CAL hydrogenation. CDD analysis (Figure S7, Supporting Information) shows charge redistribution in the Au@Au_3_Pd interface region, revealing substantial electron accumulation on the Au_3_Pd shell. This migration of electron density from the Au core, further quantified by planar‐averaged differential charge density (DCD) profiles (**Figure** **4**A), generates a Pd‐based surface enriched in electron density, which is crucial for strong adsorbate interactions.^[^ ^40^ ^]^ Work function (WF) calculations (Figure 4B) demonstrate that the intermediate WF of Au@Au_3_Pd can facilitate efficient hot electron transfer from the LSPR excited Au core to both the Pd‐based shell and the adsorbed molecules under visible light, thereby driving hydrogenation.^[^ ^41^ ^]^

Projected density of states (PDOS) analysis (Figure 4C–E) reveals the electronic complementarity of Au and Pd within the Au@Au_3_Pd catalyst. Au 5*d* orbitals serve as a deep‐lying electron reservoir, while Pd 4*d* orbitals, located near the Fermi level (E_F_), act as primary catalytic centers.^[^ ^42^ ^]^ The strong orbital overlap between Au 5*d* and Pd 4*d* bands confirms robust electronic coupling, essential for efficient plasmon‐enhanced catalysis (LSPR hot electrons transfer from Au to the Au_3_Pd shell). Further analysis of the PDOS for the Pd 4*d* (Figure 4D) and Au 5*d* (Figure 4E) bands in Au (111), Pd (111), and Au@Au_3_Pd (111) models as well as the overall electronic structure (Figure 4F) revealed an upshift in the *d*‐band center (d_c_) of Pd 4*d* compared to pure Pd (Figure 4D). Conversely, the Au 5*d* bands in both the core and shell regions of the Au@Au_3_Pd model exhibit a downshift away from E_F_ compared to pure Au (111) (Figure 4E). Notably, the Pd 4*d* *d*‐band center (d_c_) upshifting toward E_F_ suggests optimized electronic properties for reactant binding and activation at the Pd sites. This *d*‐band upshift, together with hot electron transfer from the Au core under LSPR excitation, synergistically boosts the catalytic activity of the Pd‐based shells.^[^ ^43^ ^]^ We note that the *d*‐band upshift and its catalytic relevance for the same Au@Au_3_Pd architecture were experimentally confirmed (XPS) X‐ray photoelectron spexctroscopy, and DFT in our previous study,^[^ ^33^ ^]^ where DFT‐predicted *d*‐band shifts correlated strongly with electrocatalytic trends. This provides a solid experimental basis for the electronic structure model used here. Overall, our DFT calculations highlight that the core–shell configuration of Au@Au_3_Pd NPs fosters a unique electronic environment—one that promotes plasmon‐driven hot electron transfer and optimizes Pd active sites,ultimately leading to the high efficiency observed in plasmonic‐catalytic cinnamaldehyde hydrogenation.

To bridge the experimental evidence of plasmon‐enhanced selectivity with a molecular‐level understanding of the catalytic transformation, we now turn to DFT‐based mechanistic analysis. While plasmonic excitation via LSPR provides the energetic input, through hot electron generation, it is the unique electronic structure of the Au@Au_3_Pd catalyst that determines how this energy is channeled to drive specific reaction pathways. The following analysis focuses on elucidating how this bimetallic surface selectively enables the full CAL‐to‐PPR transformation, thus mechanistically linking plasmonic excitation to selective catalysis.

**Figure 5:**
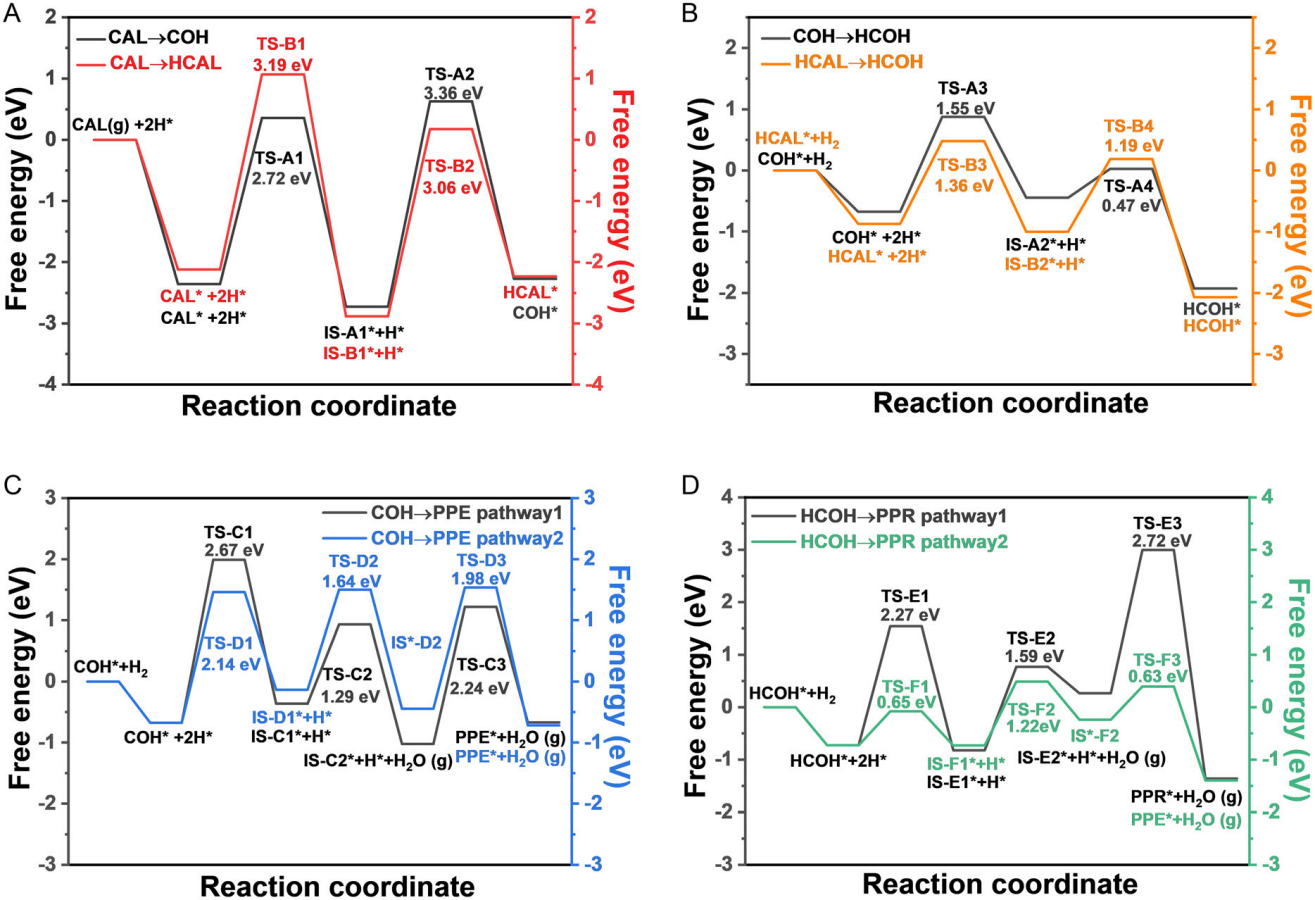
Energy landscape for selective cinnamaldehyde hydrogenation on Au@Au_**3**_Pd for different reaction pathways. A) Initial CAL hydrogenation: comparison of C=O versus C=C bond hydrogenation pathways leading to COH and HCAL, respectively. B) Energy profile for the common intermediate formation: hydrogenation of both COH and HCAL to HCOH. C) Energy profiles for alternative routes from COH to PPE. D) Energy profiles for alternative routes from HCOH to PPR.

DFT was employed to gain mechanistic insights into the observed selectivity and reaction pathways for CAL hydrogenation on the Au@Au_3_Pd catalyst, as visualized in the free energy profiles of **Figure** **5**A–D and corresponding mechanisms in Figure S8A–D, Supporting Information. While a comparative free energy analysis on bare Pd(111) was considered, we concluded that it would not meaningfully advance the mechanistic understanding of PPR formation. Experimentally, the Pd/SiO_2_ catalyst produced only partially hydrogenated products (HCAL and HCOH) with 0% PPR selectivity (Figure 2E). This aligns with our DFT adsorption analysis (Figure 2C), which shows that CAL prefers to adsorb on Pd(111) via the C=C bond, steering the reaction away from the C=O hydrogenation pathway needed for PPR. These findings already establish Pd(111) as a mechanistic dead‐end under the studied conditions. In contrast, the Au@Au_3_Pd surface reshapes the reaction landscape, enabling both C=O and C=C hydrogenation and facilitating downstream hydrogenolysis. For this reason, we focus our free energy and transition state (TS) analysis on the Au@Au_3_Pd surface, which uniquely enables the full sequence of transformations leading to PPR.

Figure 5A compares the initial hydrogenation of cinnamaldehyde (CAL) along two competing routes: C=O bond hydrogenation leading to COH (**Pathway 1** in Scheme 1) and C=C bond hydrogenation forming HCAL (**Pathway 2** in Scheme 1). The computed energy barriers reveal a slightly lower activation energy for C=C hydrogenation (TS‐B1, 3.19 eV) compared to C=O hydrogenation (TS‐A2, 3.36 eV). Although this small difference kinetically favors the formation of HCAL initially, the relatively similar activation energies suggest that both pathways are accessible from the outset,^[^ ^1^ ^]^ which is consistent with the experimental observation of the formation of both COH and HCAL within the first 4 h of reaction (Figure 3A). The subsequent temporal evolution of COH and HCAL concentrations in Figure 3A further supports the DFT predictions: the sharper decrease in COH concentration after 4 h, contrasted with HCAL reaching a maximum at 7 h before a more gradual decline until 17 h, suggests that COH is formed more rapidly and thus can be more rapidly converted to downstream products compared to HCAL, a trend reflected in the relative barrier heights in Figure 5A. The subsequent hydrogenation of both COH and HCAL to the common HCOH proceeds with facile kinetics, as evidenced by the low and similar activation energies in Figure 5B. This is corroborated by the steady increase in HCOH concentration observed experimentally over time (Figure 3A), as HCOH accumulates from both initial pathways. In contrast, the transformation of COH to PPE, as explored in Figure 5C, exhibits significantly higher energy barriers relative to COH hydrogenation to HCOH. This higher barrier is consistent with the experimentally observed low concentrations of PPE throughout the reaction (Figure 3A), indicating that this pathway is not kinetically competitive under the studied conditions. Finally, Figure 5D examines the conversion of HCOH to PPR via two pathways with distinct hydrogenolysis sequences, revealing that these final steps possess relatively low activation energies. These low barriers suggest that once HCOH is formed, its subsequent conversion to PPR is not kinetically limiting.^[^ ^44^ ^]^ Instead, the overall product selectivity is predominantly governed by the extent of hydrogenation and the progression of the hydrogenolysis steps.

**Figure 6:**
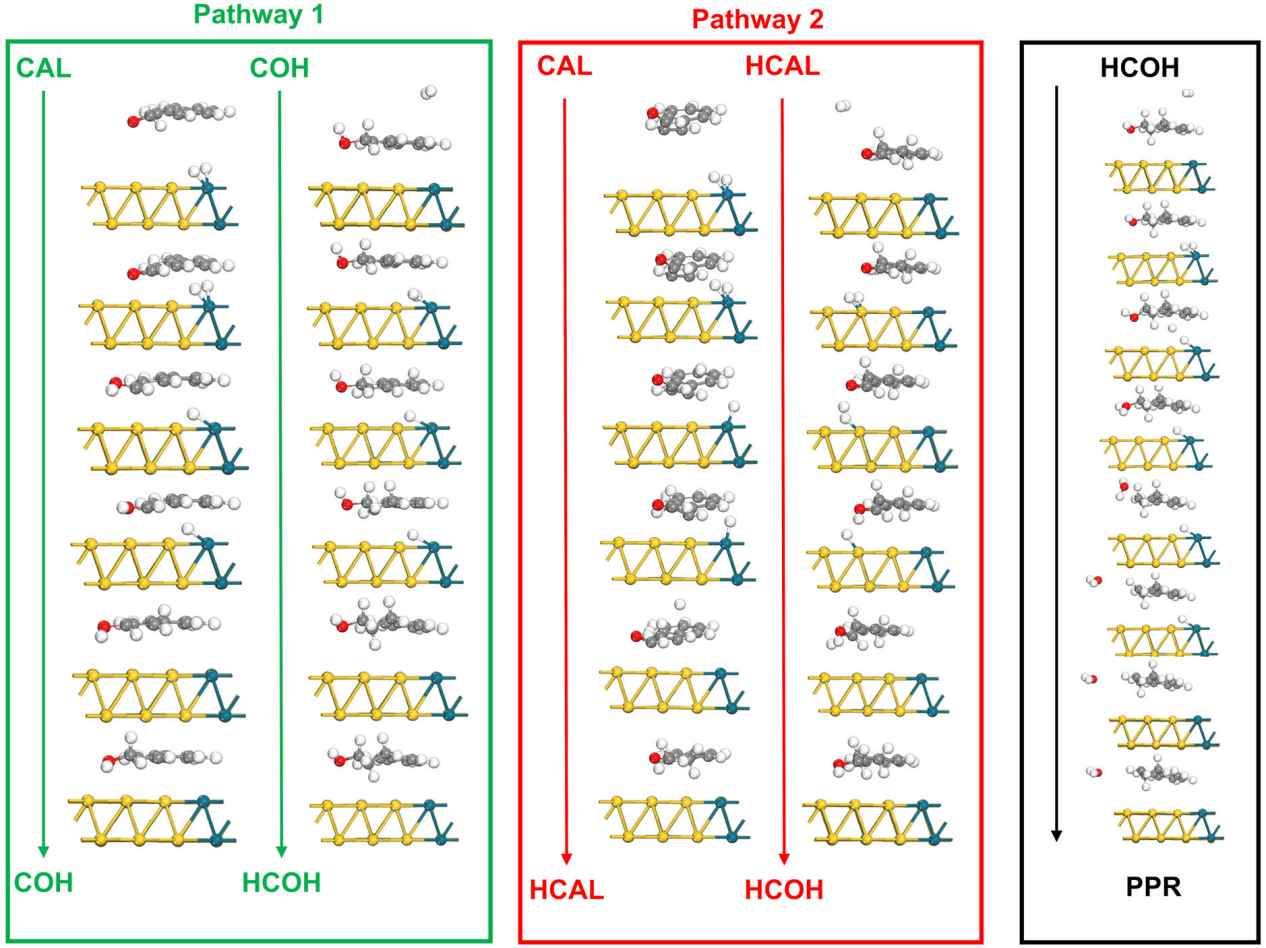
Visual summary of the proposed mechanism for Au@Au_**3**_Pd‐catalyzed cinnamaldehyde hydrogenation. Schematic overview of the key mechanistic steps. The diagram illustrates two initial hydrogenation pathways: Pathway 1 (C=O hydrogenation to COH) and pathway 2 (C=C hydrogenation to HCAL), both originating from CAL. The subsequent hydrogenation of both COH and HCAL to the common intermediate HCOH is also depicted. Finally, the HCOH hydrogenolysis step, leading to PPR, is shown as the final step, visually summarizing the complete reaction sequence on the Au@Au_3_Pd catalyst surface.

**Figure** **6** visually summarizes the mechanistic insights into cinnamaldehyde hydrogenation on the Au@Au_3_Pd catalyst surface, emphasizing the initial hydrogenation and subsequent key transformations. The mechanism initiates with cinnamaldehyde (CAL) adsorption, followed by either pathway 1 (C=O hydrogenation to COH) or pathway 2 (C=C hydrogenation to HCAL). Figure 6 also illustrates the facile convergence of both pathways at the central intermediate, HCOH, formed from both COH and HCAL hydrogenation. The final step, HCOH hydrogenolysis to PPR, is also depicted, which occurs via the transfer of H to adsorbed HCOH before the elimination of water (green pathway shown in Figure 5D). Here, LSPR‐generated hot carriers (hot electrons) and the alloying‐induced changes to electronic structure at the Pd sites could contribute to enhance the H* transfer steps. Figure 6 visually summarizes these key mechanistic steps derived from DFT, highlighting the initial hydrogenation pathways converging to PPR, the key surface intermediates involved, and illustrating how the Au@Au_3_Pd catalyst directs the complex hydrogenation network toward selective PPR formation.

## 3 Conclusions

This study demonstrates a plasmonic‐catalytic strategy for achieving control over the selectivity in the hydrogenation of cinnamaldehyde (CAL) to propylbenzene (PPR) under visible‐light irradiation. By integrating the LSPR of an Au core with the catalytic activity of a ≈1 nm thick Au_3_Pd alloy shell (25 at% Pd), our Au@Au_3_Pd/SiO_2_ catalyst achieves complete CAL conversion and a 34% PPR yield, corresponding to a 7.7‐fold increase in TOF under light‐driven conditions. Combined experimental and DFT studies reveal that LSPR‐generated hot electron transfer enhances catalytic turnover, while the bimetallic shell optimizes Pd electronic states, facilitating both C=C and C=O hydrogenation, followed by efficient hydrogenolysis to PPR. These synergistic effects overcome intrinsic selectivity limitations in α,β‐unsaturated aldehyde hydrogenation, enabling a level of control not achievable with the thermal catalysis counterpart. Beyond demonstrating selectivity, stability, and reusability, this work validates a plasmonic‐catalytic design principle that harnesses light excitation and bimetallic tuning to drive challenging hydrogenation reactions under mild conditions. This approach provides a foundation for designing next‐generation plasmonic catalysts, offering a versatile platform for sustainable and selective molecular transformations. Looking ahead, the insights gained here open new directions for light‐driven catalysis, with significant potential for industrial applications in fine chemicals, pharmaceuticals, and renewable energy technologies. While the present study focused on elucidating plasmon‐enhanced selectivity under defined mild conditions, future work will aim to explore a broader operational window, including variations in hydrogen pressure and temperature, to further optimize catalytic performance and evaluate scalability.

## 4 Experimental Section

4.1

4.1.1

##### Materials and Methods

All reagents used in this study were of analytical grade and employed without further purification. Chloroauric acid trihydrate (HAuCl_4_·3H_2_O, 99.9%, Sigma‐Aldrich), sodium citrate trihydrate (99%, Sigma‐Aldrich), L‐ascorbic acid, potassium hexachloropalladate (IV) (K_2_PdCl_6_, 99%, Sigma‐Aldrich), nitric acid (70%, Sigma‐Aldrich), fumed silica (WACKER HDK T40), isopropanol (IPA, HPLC grade, Sigma‐Aldrich), cinnamaldehyde (99%, Sigma‐Aldrich), cinnamyl alcohol (95%, Alfa Aesar), hydrocinnamaldehyde (98%, Sigma‐Aldrich), hydrocinnamyl alcohol (98%, Sigma‐Aldrich), and phenylpropane (98%, Sigma‐Aldrich) were used as substrates and/or standards. Hydrogen gas (>99.9% purity) was utilized in catalytic experiments. Deionized (DI) water (18.2 MΩ·cm) was used throughout all procedures.

UV−vis spectra were acquired directly from the NP aqueous suspensions using a Shimadzu UV‐2600 spectrometer from 800 to 200 nm with a step size of 1 nm. Elemental composition analysis was performed by microwave plasma atomic emission spectroscopy (MP‐AES) using Agilent Technologies 4100 MP‐AES. Three independent measurements were performed for each sample. Transmission electron microscopy (TEM) images were acquired on a JEOL JEM‐1400 TEM operated at 120 kV. TEM samples were prepared by dispersing the NP suspension in DI water with an ultrasonic bath and drop casting onto carbon‐coated copper grids. SEM images were recorded on a field‐emission microscope (Hitachi S‐4800). The suspension containing the NPs was drop cast onto a Si wafer and dried under ambient conditions before imaging.

The high‐angle annular dark field (HAADF) STEM imaging was performed using a probe‐corrected FEI Titan G2 80−200 S/TEM instrument equipped with the Super‐X EDX detector and a Gatan Quantum ER imaging filter for electron energy loss spectroscopy. The Titan STEM was operated at an accelerating voltage of 200 kV. The HAADF STEM images were acquired using a probe current of 300 pA, convergence semiangle of 21.5 mrad, and a HAADF inner collection angle of 43 mrad. The images were collected with a dwell time of 10 μs, resulting in a total frame time per image of ≈12.6 s. The STEM‐EDX spectrum images were collected with a probe current of 300 pA using all 4 EDX detectors for a total acquisition time of ≈10 min per dataset. Analysis of the STEM‐EDX data was carried out using in‐house built Python scripts with packages including Hyperspy v1.6.31.^[^ ^45^ ^]^

##### Synthesis of Au and Au@Au3Pd NPs

Gold nanospheres were synthesized via citrate reduction.^[^ ^46^ ^]^ In a typical procedure, 100 mg of sodium citrate trihydrate was dissolved in 148 mL of DI water in a 250 mL round‐bottom flask. The solution was heated to 100 °C under magnetic stirring for 15 min, followed by the rapid addition of 2 mL of an aqueous HAuCl_4_ solution (12.7 mM). The reaction proceeded for 30 min, yielding a red suspension of Au NPs.

To synthesize Au@Au_3_Pd core–alloyed shell NPs,^[^ ^33^ ^]^ 75 mL of freshly prepared Au NP solution was mixed with 35.2 mg of L‐ascorbic acid (0.2 mmol) and stirred at 70 °C for 30 min in a 150 mL round‐bottom flask. Aqueous K_2_PdCl_4_ (1 mg mL^−1^) was then added in varying volumes (21.5 μL, 107 μL, and 215 μL) to obtain Au_99.7_Pd_0.3_, Au_98.5_Pd_1.5_, and Au_97_Pd_3_ NPs, respectively, in terms of overall at.%. The reaction was allowed to proceed for 30 min to ensure complete deposition of Pd, with STEM characterization of all systems suggesting the presence of Pd as a ≈1 nm thick Au_3_Pd alloyed shell.

##### Preparation of the Supported Au@Au_3_Pd/SiO_2_ Catalysts

For catalyst immobilization, 20 μL of nitric acid was added to 75 mL of freshly prepared Au_99.7_Pd_0.3_, Au_98.5_Pd_1.5_, and Au_97_Pd_3_ NP suspensions. Subsequently, 80 mg of dried commercial SiO_2_ (fumed silica) was introduced, and the mixture was stirred at 70 °C for 24 h. The resulting material was separated by centrifugation, washed three times with DI water, and dried at 70 °C overnight.

##### Photocatalytic Activity Tests

The catalytic hydrogenation of cinnamaldehyde was conducted in a 100 mL Fisher‐Porter glass reactor equipped with a pressure regulator. In a typical experiment, 30 mg of catalyst was dispersed in 10 mL of isopropanol and sonicated for 10 min. Cinnamaldehyde (1.6 mmol) was then added. The reactor was sealed, purged three times with nitrogen (6 bar) and three times with hydrogen (6 bar), and subsequently pressurized to 6 bar with hydrogen. The reaction mixture was stirred at 70 °C under 525 nm LED irradiation with four Kessil PR 160 L LED lamps (total irradiance of 59.50 mW cm^−2^ per lamp) for 24 h. The 427 nm lamp had a total irradiance of 126.1 mW cm^−2^ per lamp. The setup used for LSPR excitation was made of four 425 nm LED lamps equally spaced around the reactor, at a distance of 7 cm. The reactor was positioned in the center of the system, immersed in an oil bath over a temperature‐controlled magnetic stirrer. This enables control over the temperature and more uniform illumination of the reaction mixture from all directions.^[^ ^47^ ^]^ All the catalytic tests were performed in triplicate and the average data is presented. After the reaction, products were separated by centrifugation and analyzed by gas chromatography (GC) using a Shimadzu NEXIS GC‐2030 equipped with a flame ionization detector and a Crossbond SH‐Rxi5ms column (30 m × 0.24 mm × 0.25 μm). Product identification was performed using an Agilent HP5977A mass spectrometer connected to an Agilent 7890B GC with an HP‐5MS column. The cinnamaldehyde conversion (%) was calculated using the following Equation (1).

(1) $$\text{Conv }\left(\%\right)=\frac{A_{t0}-A_{t}}{A_{t0}}\times \;100$$

where *A*_*t*0_ and *A*_*t*_ are the chromatographic area of cinnamaldehyde in the samples at *t* = 0 and at time *t* = *t*, respectively. The product selectivity was calculated using Equation (2).

(2) $$S_{i}\left(\%\right)=\frac{A_{i}}{\sum A_{j}}\times 100$$

where *A*_*i*_ is the chromatographic area of product *i* and ∑ *A*_*j*_ is the sum of the chromatographic area of all detected products. The TOF was calculated according to Equation (3).

(3) $$\text{TOF}=\;\frac{mol_{conv}}{mol_{Metal}\times \;t}$$

where *mol*_conv_ is the moles of CAL converted, *mol*_Metal_ is the moles of the active sites (Pd), and *t* is the reaction time in hours.

##### Computational Methods

DFT calculations were performed with the first‐principles simulation, cambridge sequential total energy package (CASTEP) module in Materials Studio software.^[^ ^48^ ^]^ The exchange‐correlation potential was described by the generalized gradient approximation (GGA) with the Perdew–Burke–Ernzerhof (PBE) functional.^[^ ^49^ ^]^ The interactions between valence electrons and ionic cores were described by the on‐the‐fly generated (OTFG) ultrasoft pseudo‐potential method. A plane‐wave basis set with a cutoff energy of 380 eV was assigned to the potential method. The empirical dispersion correction in Grimme's scheme was employed to consider the van der Waals (vdW) interaction.^[^ ^50^ ^]^ The Broyden–Fletcher–Goldfarb–Shannon algorithm with a medium quality setting of k‐points was used for all the energy minimizations in this work. The geometry optimization convergence tolerances for the energy change, maximum force, and maximum displacement were 5 × 10^−5^ eV atom^−1^, 0.001 eV Å^−1^, and 0.005 Å, respectively. For all the models, a 20 Å vacuum space was set in the z‐axis to guarantee full relaxation. The TS search was conducted by the Complete LST/QST protocol in materials studio.^[^ ^51^ ^]^

The adsorption energies (*E*ads) at the various surface sites were calculated using Equation (4).

(4) $$E_{\text{ads}}\;=\;E_{A/B}\;-\;E_{A}\;-\;E_{B}$$

where *E*_A/B_, *E*_A_, and *E*_B_ are the total energies of the adsorbed system, the free adsorbate, and the surface model. The free energy (Δ*G*) calculations of each elementary step were based on the standard hydrogen electrode model.^[^ ^52^ ^]^ The reaction free energy change can be obtained with Equation (5).

(5) $$\Delta G\;=\text{ Δ}E+\Delta E_{\text{ZPE}}-T\Delta S\;$$

where Δ*E* is the total energy difference before and after the intermediate is adsorbed, and Δ*E*_ZPE_ and Δ*S* are, respectively, the differences of zero‐point energy and entropy.

## Conflict of Interest

The authors declare no conflict of interest.

## Supporting information

Supplementary Material

## Data Availability

The data that support the findings of this study are available in the supplementary material of this article.
