# Supplementary material for: Plasmonic Catalysis for Controlling Selectivity in the Hydrogenation of Cinnamaldehyde to Propylbenzene Under Visible‐Light Irradiation
Source: ChemSusChem. 2025 Aug 12;18(19):e202501054. doi: 10.1002/cssc.202501054 (PMC12487739; doi:10.1002/cssc.202501054)
Supplement: Supplementary file 1 — Supplementary Material [file CSSC-18-e202501054-s001.pdf]

Supporting Information for

Plasmonic Catalysis for Controlling Selectivity in the Hydrogenation of Cinnamaldehyde to Propylbenzene under Visible Light Irradiation

Sana Frindy,[1,†] Shiqi Wang,[1,†] Sam Sullivan−Allsop,[2] Rongsheng Cai,[2] Thomas J. A. Slater,[3] Sarah J. Haigh,[2] and Pedro H. C. Camargo[1,*]

[1]*Department of Chemistry, University of Helsinki, A.I. Virtasen aukio 1, PO Box 55, FIN-0014 Helsinki, Finland*

[2]*Department of Materials, University of Manchester, Manchester M13 9PL, United Kingdom*

[3]*Cardiff Catalysis Institute, School of Chemistry, Cardiff University, Cardiff CF10 3AT, United Kingdom*

**Corresponding author. Email: pedro.camargo@helsinki.fi*

[†]These two authors contributed equally to this work.

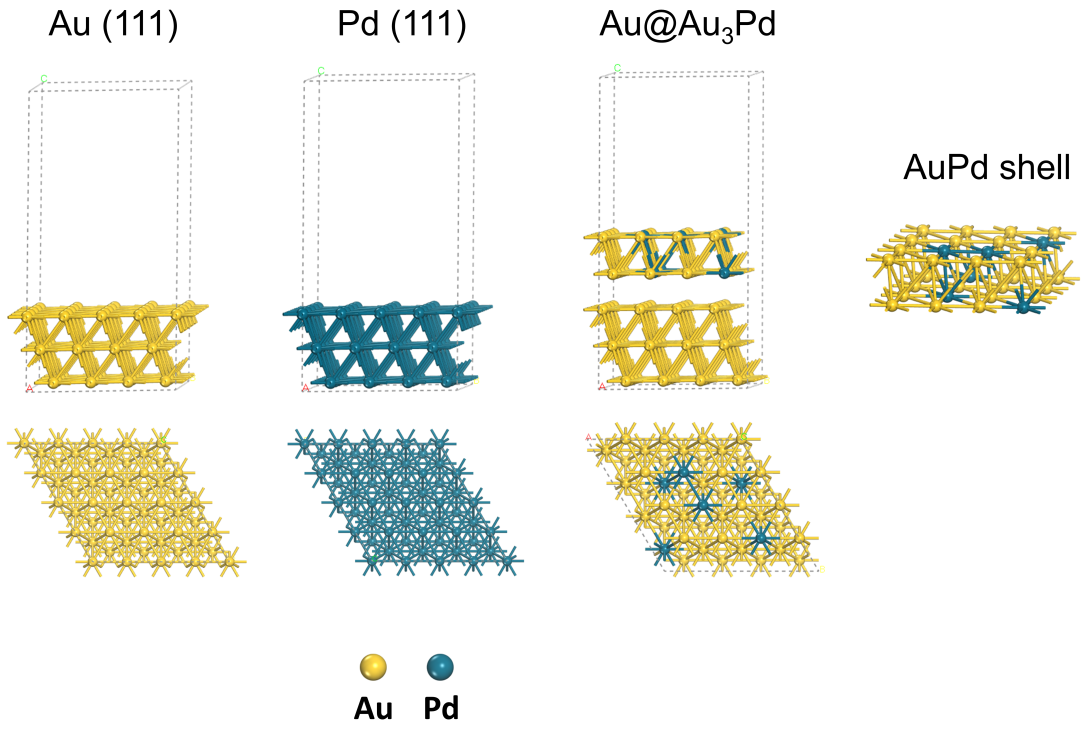

Figure S1. Atomistic Models for DFT Calculations of Catalytic Surfaces. Schematic representations of the surface models employed in DFT calculations. (Left) Model of the Au(111) surface. (Center) Model of the Pd(111) surface. (Right) Model of the Au@Au_3Pd core-shell nanoparticle (NP) surface. In the Au@Au_3Pd NP model, the core is composed of gold (Au), while the shell consists of an Au_3Pd alloy. In all models, Au atoms are depicted in yellow and Pd atoms are shown in blue.

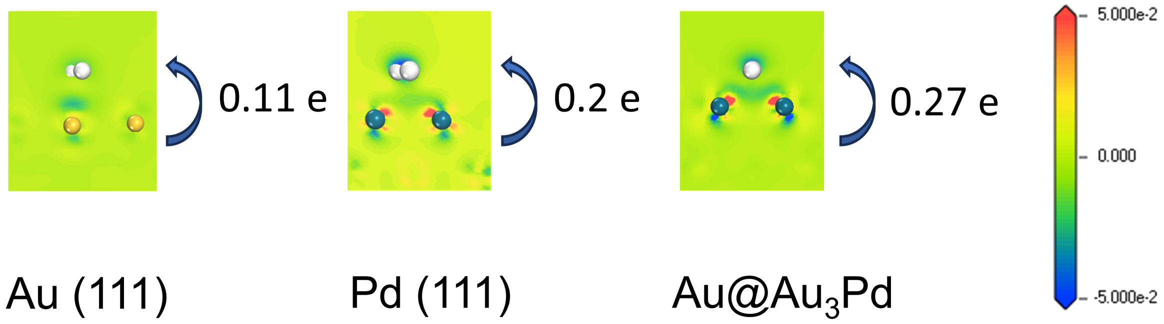

Figure S2. Charge Density Difference and Mulliken Charge Analysis of H_2 Adsorption. Two-dimensional charge density difference (CDD) plots and corresponding Mulliken charge analysis for H_2 molecule adsorbed on (Left) Au(111), (Center) Pd(111), and (Right) $Au@Au_3Pd$ surfaces. CDD plots visualize the redistribution of electron density upon H_2 adsorption, with blue regions indicating electron depletion and red regions indicating electron accumulation. The isosurface level for the CDD plots is set to ±0.005 e $Å^{-3}$. Mulliken charge values (in e) for the adsorbed H_2 molecule are displayed above each corresponding CDD plot, quantifying the electron density transfer between the surface and H_2 molecule. The color scale for CDD plots ranges from blue (-0.05 e $Å^{-3}$) to red (+0.05 e $Å^{-3}$).

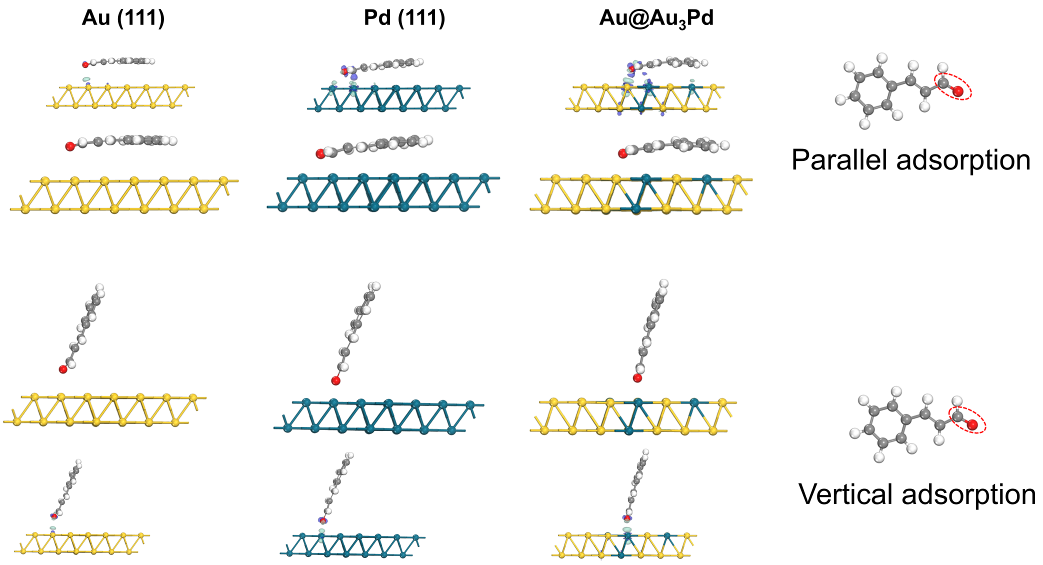

Figure S3. Charge Density Difference Analysis of Cinnamaldehyde (CAL) C=O Bond Adsorption. Two-dimensional charge density difference (CDD) plots illustrating the electronic perturbation upon cinnamaldehyde (CAL) adsorption via the carbonyl (C=O) bond on: Au (Left Column), Pd (Center Column), and $Au@Au_3Pd$ surfaces. (Top Row) Parallel adsorption configurations, where the C=O bond is oriented approximately paralell to the surface. (Bottom Row) Vertical adsorption configurations, where the C=O bond is oriented approximately perpendicular to the surface. In CDD plots, red regions indicate electron accumulation and blue regions indicate electron depletion (isosurface level: ±0.005 e $Å^{-3}$; color scale: -0.05 to 0.05 e $Å^{-3}$).

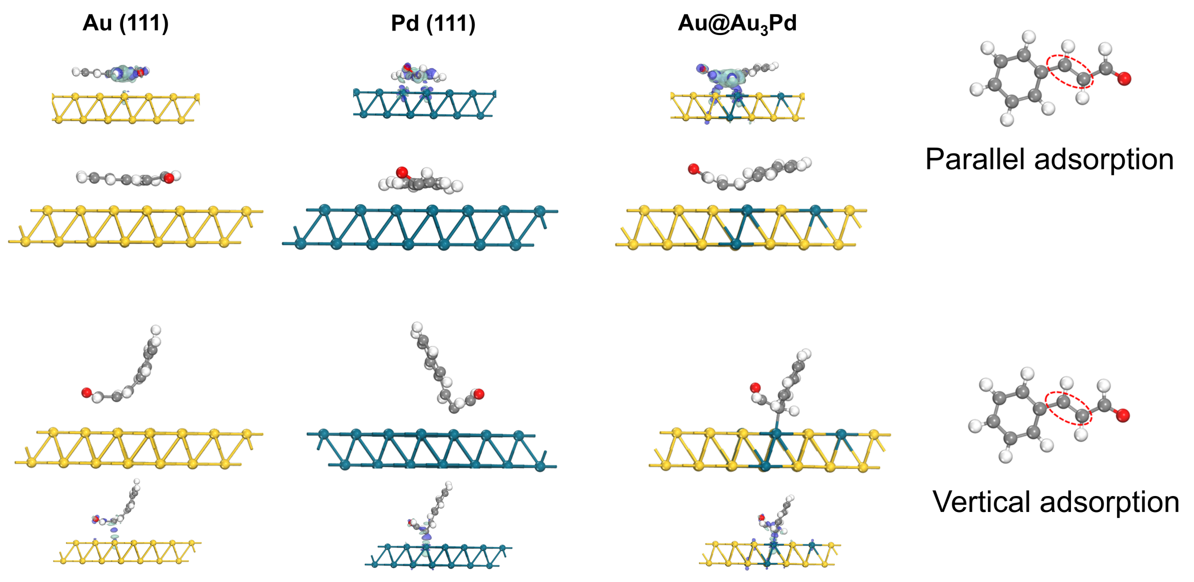

Figure S4. Charge Density Difference Analysis of Cinnamaldehyde (CAL) C=C Bond Adsorption. Two-dimensional charge density difference (CDD) plots illustrating the electronic perturbation upon cinnamaldehyde (CAL) adsorption via the carbon-carbon double (C=C) bond on Au (Left Column), Pd (Center Column), and Au@Au_3Pd (Right Column) surfaces. (Top Row) Parallel adsorption configurations, where the C=C bond is oriented approximately parallel to the surface. (Bottom Row) Vertical adsorption configurations, where the C=C bond is oriented approximately perpendicular to the surface. In CDD plots, red regions depict electron accumulation, and blue regions depict electron depletion (isosurface level: ±0.005 e $Å^{-3}$; color scale: -0.05 to 0.05 e $Å^{-3}$).

Table S1. Catalytic Performance of Au/SiO_2, Pd/SiO_2, and Au@Au_3Pd/SiO_2 Catalysts in Cinnamaldehyde Hydrogenation. Comparison of catalytic activity and product distribution for cinnamaldehyde (CAL) hydrogenation over Au/SiO_2, Pd/SiO_2, and Au@Au_3Pd/SiO_2 catalysts. The table presents cinnamaldehyde (CAL) conversion (%), product yields for cinnamyl alcohol (COH), hydrocinnamyl alcohol (HCOH), hydrocinnamaldehyde (HCAL), and propylbenzene (PPR), and turnover frequency (TOF) for PPR formation (TOF PPR, h^{-1}). Reactions were conducted under 525 nm light irradiation in isopropanol solution. "-" indicates "not detected". Control experiments in which the Pd loading in the nanoparticles was decreased from 3 at.% in Au@Au_3Pd/SiO_2 to 1.5 and 0.3 are also shown (Au@Au_7Pd/SiO_2 and Au@$Au_{39}Pd$/SiO_2, respectively).

Catalyst	Pd at% in NPs	Pd catalyst loading (wt. %)	% Conv	Yield%				TOF PPR (h^{-1})
				COH	HCOH	HCAL	PPR	
**Au-SiO_2**	-	-	-	-	-	-	-	-
**Pd-SiO_2**	100	3	100	-	19	81	-	-
**Au@Au_3Pd/SiO_2**	3	0.05	100	8	55	3	34	$16.4 \cdot 10^3$
**Au@Au_7Pd/SiO_2**	1.5	0.032	100	-	58	33	9	$3.6 \cdot 10^3$
**Au@$Au_{39}Pd$/SiO_2**	0.3	0.005	100	-	36	62	2	$1.2 \cdot 10^3$

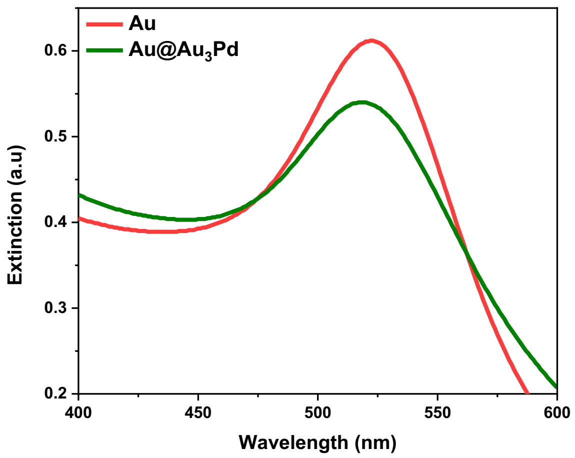

Figure S5. **Optical properties.** UV-Vis extinction spectra recorded from aqueous suspensions containing Au (red trace) and Au@Au_3Pd (green trace) NPs.

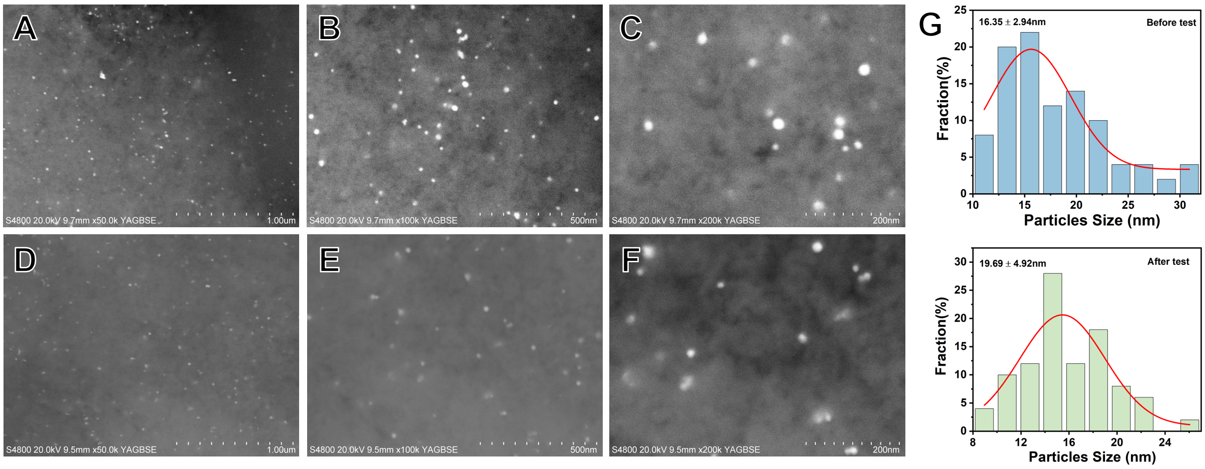

Figure S6. Morphology Retention of Au@Au_3Pd/SiO_2 Catalyst Upon Reuse. SEM images of the (A-C) fresh and (D-F) spent Au@Au_3Pd/SiO_2 catalyst. (G) Histograms of size distribution for the (top) fresh and (bottom) spent Au@Au_3Pd/SiO_2 catalyst. The NP sizes were 16 $\pm$ 3 and 20 $\pm$ 5 nm, respectively.

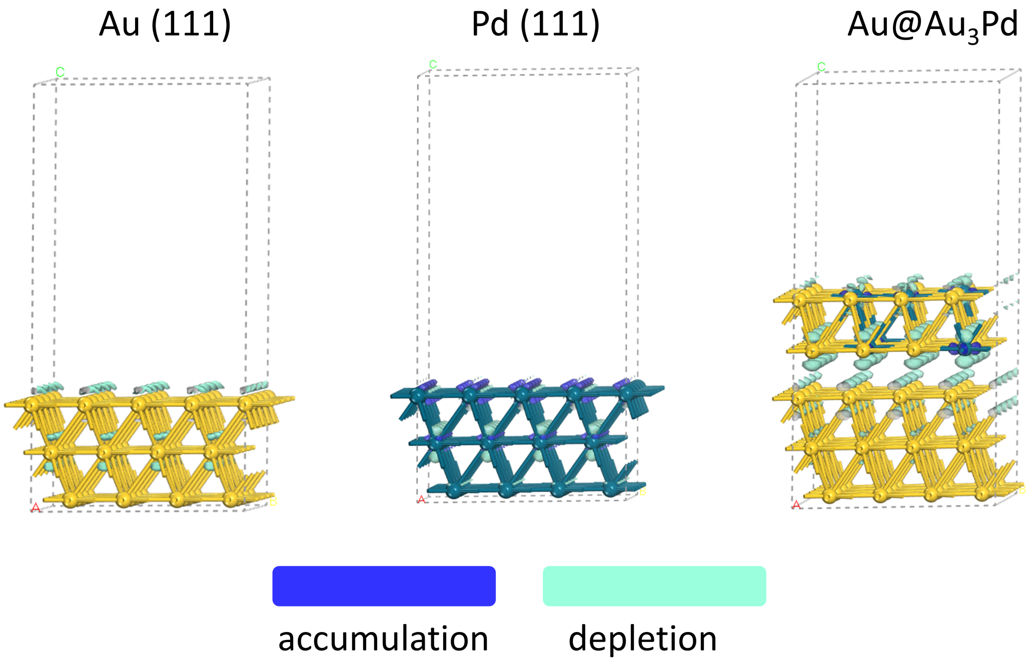

Figure S7. Charge Density Difference Analysis of Au(111), Pd(111), and Au@Au$_3$Pd Surface Models. Two-dimensional charge density difference (CDD) plots visualizing the intrinsic electron density redistribution upon the formation of different surface models: Au (Left), Pd (Center), and Au@Au$_3$Pd (Right). In CDD plots, bright green contours represent regions of electron depletion, and blue contours represent regions of electron accumulation, relative to a superposition of atomic electron densities. The isosurface level for the CDD plots is set to ±0.005 e Å^{-3}.

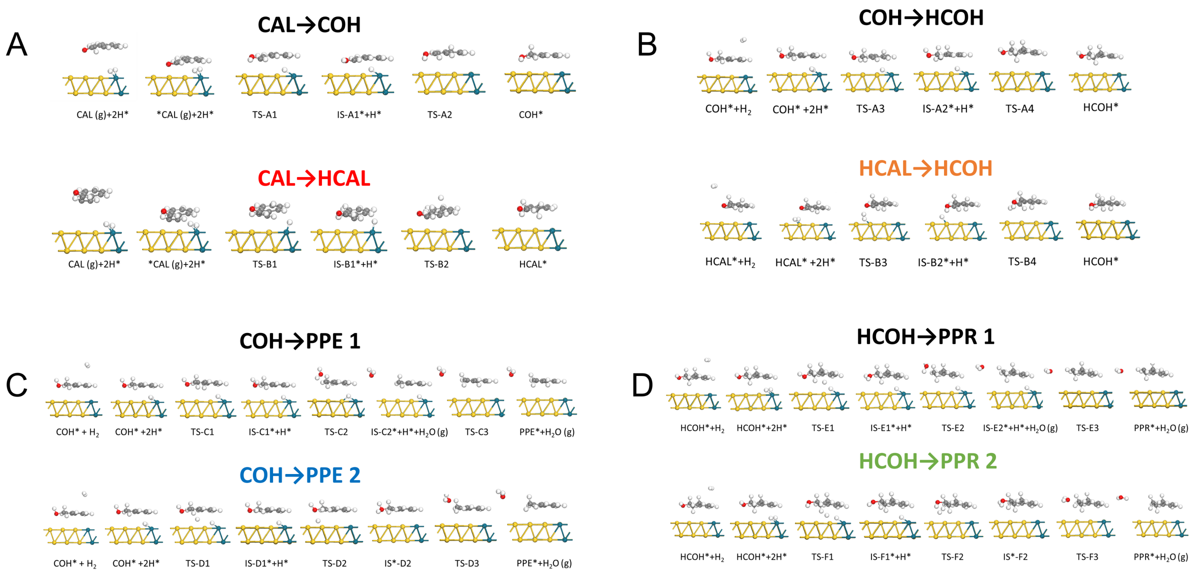

Figure S8. Visual Mechanistic Summary of Au@Au₃PdCatalyzed Cinnamaldehyde Hydrogenation. This figure schematically summarizes the proposed reaction mechanism for cinnamaldehyde hydrogenation over the Au@Au_3Pd catalyst. (A) Initial hydrogenation pathways: C=O and C=C bond hydrogenation leading to cinnamyl alcohol (COH) and hydrocinnamaldehyde (HCAL), respectively. (B) Hydrogenation of intermediates: Conversion of both COH and HCAL to hydrocinnamyl alcohol (HCOH). (C) Alternative routes from COH: Pathways from cinnamyl alcohol (COH) to 1-phenylpropene (PPE). (D) Hydrogenolysis pathways: Conversion of hydrocinnamyl alcohol (HCOH) to propylbenzene (PPR).
